# Nanoindentation under retrocorneal pressure to determine the biomechanical response of the human cornea to accelerated UVA crosslinking and riboflavin osmolarity ex vivo

**DOI:** 10.1038/s41598-025-24522-6

**Published:** 2025-10-21

**Authors:** Robert Lohmüller, Günther Schlunck, Bero Bressler, Daniel Böhringer, Thomas Reinhard, Stefan J. Lang

**Affiliations:** 1 Eye Center, Medical Center, Faculty of Medicine, University of Freiburg, Killianstr. 5, 79106 Freiburg im Breisgau, Germany; 2 Department of Ophthalmology, University Hospital Brandenburg, Brandenburg Medical School, Brandenburg an Der Havel, Germany

**Keywords:** Corneal crosslinking, Accelerated protocols, Riboflavin osmolarity, Corneal biomechanics, Nanoindentation, Intraocular pressure, Biological models, Biological physics, Biological techniques, Biophysics, Structural biology

## Abstract

Riboflavin-mediated UVA corneal crosslinking (CXL) is an established treatment to halt the progression of keratoconus. However, the biomechanical effects of accelerated protocols and varying riboflavin osmolarities under physiological conditions remain poorly understood. Traditional biomechanical assessments using nanoindentation typically analyze tissue flatmounts, disregarding the natural tissue strain exerted by intraocular pressure. We developed a novel experimental setup combining nanoindentation with adjustable retrocorneal fluid pressure (RCP) to simulate physiological conditions during measurements. Using this approach, we first examined five corneas under incrementally increasing RCP levels to quantify the effect of tissue pre-tension on biomechanical properties. In a subsequent series, we investigated 70 human corneas to evaluate seven different protocols, including accelerated treatment protocols and different riboflavin osmolarities, by analyzing the Hertz-elastic modulus (E_HZ_) and creep behavior (C_IT_). Our results demonstrate that all CXL protocols significantly enhanced corneal stiffness and reduced tissue creep. Compared to the standard Dresden protocol, the biomechanical effect was enhanced with hyperosmolar riboflavin but diminished when high radiation intensity was used for shorter duration. Furthermore, increasing RCP led to non-linear changes in corneal biomechanics, manifesting as increased E_HZ_ and decreased C_IT_. These findings emphasize the importance of considering physiological tissue pre-tension in biomechanical assessments and provide new insights for optimizing CXL treatment protocols.

## Introduction

Understanding human corneal biomechanics is crucial for gaining deeper insights into corneal ectatic pathologies such as keratoconus. These conditions, characterized by progressive corneal thinning and structural alterations, significantly impact vision and corneal integrity^1^. The development of riboflavin-mediated UVA crosslinking (CXL) to slow down or halt the progression of keratoconus^2,3^ has further emphasized the importance of accurately assessing corneal biomechanical properties. Corneal biomechanics have been studied using various techniques. In vivo methods include non-contact tonometry with Corvis ST for air-puff-induced deformation analysis^4,5^, the Ocular Response Analyzer (ORA) which measures corneal hysteresis and corneal resistance factor^5,6^, Goldmann applanation tonometry^7^, and Brillouin microscopy^8,9^. Ex vivo approaches encompass uniaxial extension^10–12^, atomic force microscopy^13,14^, nanoindentation (NI)^15–19^ and optical coherence elastography^20–22^. Each of these techniques offers unique insights, contributing to the overall understanding of this complex tissue.

Our study introduces a novel approach to examining human corneal tissue mimicking physiological conditions. We have developed a method for sample fixation which allows to apply a well-controlled retrocorneal fluid pressure during NI measurements. This technique addresses a key limitation of traditional biomechanical assessments, which often use flatmounted corneal samples and neglect the crucial role of intraocular pressure (IOP) in corneal shape and function. By using intact human donor corneas in an artificial anterior chamber, we simulate physiological IOP during measurements. This setup allows us to study corneal biomechanics under various degrees of mechanical pre-tension, offering insights more relevant to in vivo conditions. Our NI approach enables detailed, spatially resolved characterization of corneal biomechanical properties in a near-physiological state.

CXL has seen significant advancements, focusing on enhancing treatment efficiency and patient comfort. Accelerated protocols aim to reduce corneal irradiation time, improving patient experience while potentially achieving similar stiffening effects to standard protocols, albeit with possible differences in crosslinking depth and distribution. Hypo-osmolar riboflavin solutions allow treatment of patients with thin corneas by inducing corneal swelling to achieve the necessary thickness for irradiation. Our study quantifies the effects of these CXL protocols on corneal biomechanics under near-physiological conditions, contributing to the optimization of CXL treatments. For a comprehensive overview of recent CXL advances, see Raiskup et al.^23^.

The current study comprises two main components. First, using healthy donor corneas, we aim to characterize the biomechanical properties of corneal tissue by comparing flatmounted samples with pre-tensioned tissue under physiological conditions. In particular, we assessed the influence of tissue pre-tension, generated by intraocular pressure, on corneal biomechanics. This approach not only reveals how mechanical pre-tension affects corneal biomechanics but also improves the physiological relevance and quality of the measurements. Second, we utilize this newly developed method to evaluate the treatment effects of different CXL protocols. We assess the impact of various riboflavin solutions and accelerated CXL protocols by comparing the initial biomechanical properties of the corneal tissue with those measured after each treatment. This approach allows us to quantify the changes induced by different CXL techniques under near-physiological conditions. Through these investigations, we aim to enhance the understanding of corneal biomechanics and the effects of various CXL protocols.

## Materials and methods

### Corneal tissue and preparation

Human donor corneas not suitable for transplantation due to low endothelial cell count were obtained from LIONS Cornea Bank Baden-Württemberg. Ethical approval from the Ethics Committee of the Albert-Ludwigs-University of Freiburg (408/15) was given. The standards of the Declaration of Helsinki were followed. Informed consent for corneal donation and, if applicable, for the use for research purposes was obtained from the donors or, if not available, from their next of kin.

### Initial equilibration in culture medium

**Fig. 1:**
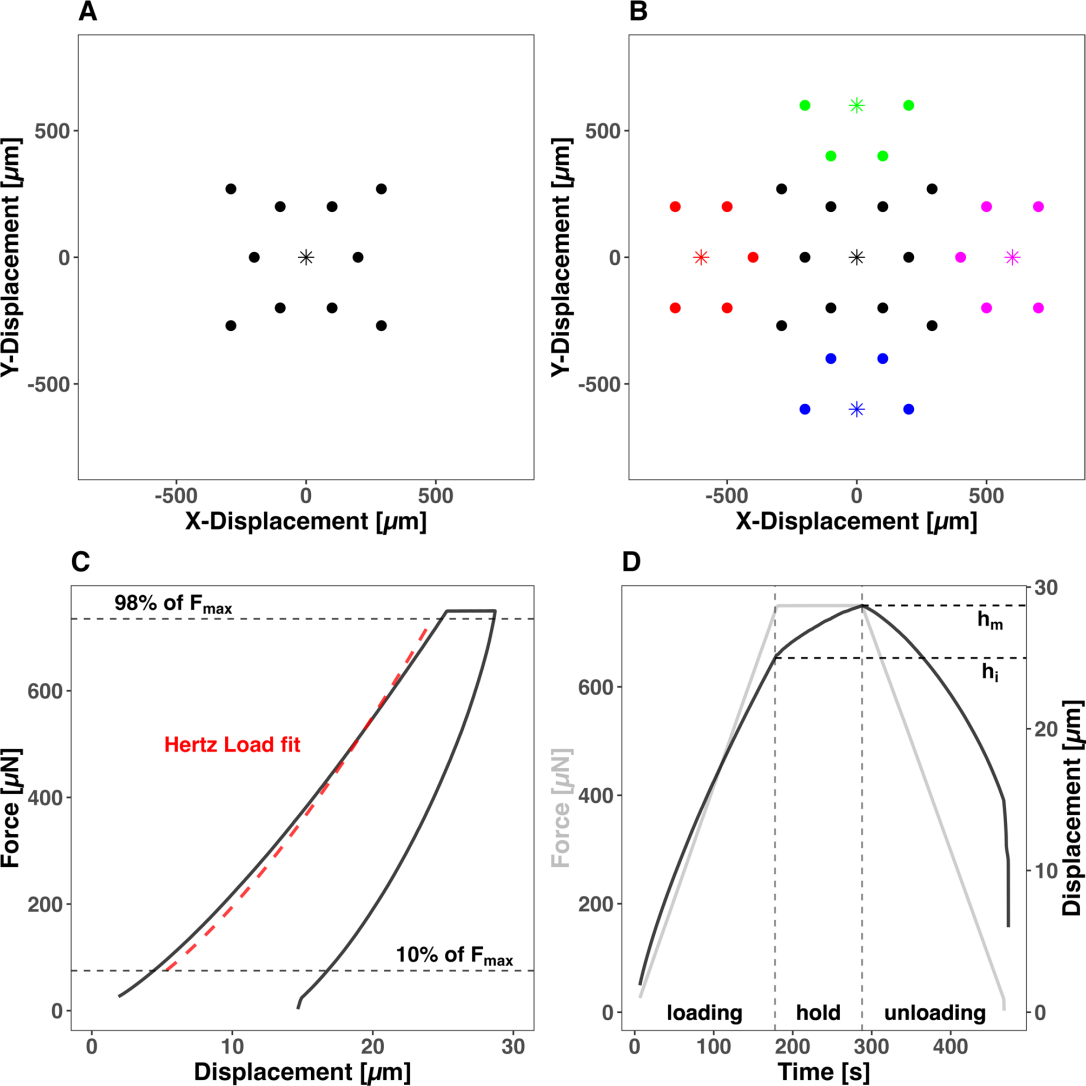
Nanoindentation (NI) matrix of 10 spatially resolved NIs (points) in the center of the corneal tissue with one surface detection using an adjust depth offset (ADO) illustrated by an asterisk (**A**). Extended Nanoindentation matrix for quantifying corneal crosslinking (CXL) effects with 28 NIs (points) assigned to a total of five ADO (asterisks) (**B**). The Force–Displacement diagram a typical progression curve of the NI with Hertz fitted loading part from 10 to 98% of the maximum Force (F_max_) applied (**C**). A typical Force–Time and Displacement–Time curve from a NI with highlighted progression of Displacement during constant load from h_i_ to h_m_ (**D**).

The corneas were equilibrated in MEM Earl’s cell culture medium (BS.F0325, Bio & SELL, GmbH, Feucht, Germany) supplemented with 15% dextran 500 (9219.1, Carl Roth GmbH + Co. KG, Karlsruhe, Germany), 10% fetal bovine serum (FBS, FBS.S 0615, Bio & SELL, GmbH, Feucht, Germany), 100 U / mL penicillin and 100 µg / mL streptomycin (P4333-100ML, Sigma-Aldrich, St. Louis, MO, USA), 2,5 mg Amphotericin B (A2942-50ML, Sigma-Aldrich, St. Louis, MO, USA), 12,5 mM HEPES-Buffer (P05-01100, PAN-Biotech GmbH, Aidenbach, Germany) and 2 mM L-Glutamine (25030-081, Life Technologies, Paisley, UK) for 24 h at + 37 °C to control the tissue hydration (Fig. 1).

### Artificial anterior chamber setup

**Fig. 2:**
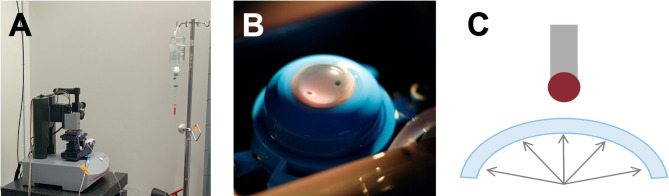
UNHT^3^ Bio Nanoindenter (Anton Paar, Graz) (**A**). Cornea clamped in an artificial anterior chamber (**B**). Schematic drawing of the experimental setup (**C**).

For NI measurements, the corneal tissue was clamped in a modified artificial anterior chamber (K20-2125, Barron Precision Instruments L.L.C., Grand Blanc, MI, USA). The chamber consists of a tissue pedestal and a matching tissue retainer, which is placed over the cornea after centration. The tissue is then securely fixed by tightening a locking ring, thereby compressing the scleral rim to achieve a watertight seal. The inner chamber of the artificial anterior chamber was filled with dextran-supplemented cell culture medium. One of the two connector tubes of the artificial anterior chamber was connected to both a saline infusion and a pressure sensor (G19237 Mod. Duesseldorf, Geuder, Heidelberg, Germany), while the second tube was clamped to close the system. The adjustable retrocorneal fluid pressure was controlled by varying the height of the connected saline infusion, and the pressure was continuously monitored by the pressure sensor. The modified artificial anterior chamber was fixed in a specifically developed 3D-printed (Form 3B + , Tough 2000 Resin, Formlabs GmbH, Berlin, Germany) sample mount for the biomechanical characterization (Fig. 2).

### Measurement conditions

All measurements were conducted with the tissue immersed in a bath of cell culture medium supplemented with 15% dextran. This approach served two purposes: preventing dehydration and maintaining corneal thickness within a physiological range. Prior to native measurements, the corneal epithelium was removed. Following CXL, each cornea was kept overnight in medium supplemented with 15% dextran to equilibrate tissue hydration. This step ensured that biomechanical properties could be measured at comparable thicknesses across samples.

### Nanoindentation

Nanoindentation (NI) was utilized in this study to assess both the elastic properties and the time-dependent deformation of corneal tissue samples. The measurements were performed using a UNHT^3^ Bio Nanoindenter (Anton Paar, Graz, Austria) equipped with a spherical ruby tip with a radius of 500 µm. During the testing protocol, the indenter applied force to the corneal tissue samples until a maximum penetration depth of 25 µm was reached. The force required to achieve this depth (F_max_) was maintained for 90 s before the unloading phase commenced. The force–displacement and time course of the indentation protocol are illustrated in Fig. 1.

### Elastic properties: the hertz elastic modulus

The elastic properties of the tissue were quantified by calculating the Hertz elastic modulus (E_HZ_) based on the force–displacement data using Eq. 1:

1 $$F=\frac{4}{3}E\sqrt{R}{(h)}^\frac{3}{2}$$

where $F$ represents the indentation force, $R$ the radius of the indenter tip, and $h$ the penetration depth. The calculation was performed using data in the range between 10 and 98% of F_max_, following methods from previous studies^15,17^.

### Time-dependent deformation: the creep index

In addition to elastic properties, NI enables the analysis of time-dependent deformation under a constant force, which reflects the viscoelastic behavior of the tissue. During the 90-s holding phase, the indentation depth gradually increases due to the material’s viscoelastic response. The creep index (C_IT_) was used to quantify this behavior and was calculated as in previous studies^15,17^ using Eq. 2:

2 $${C}_{IT}= \frac{({h}_{m}-{h}_{i})}{{h}_{m}}$$

where ${h}_{i}$ represents the initial indentation depth at the beginning of the holding period, and ${h}_{m}$ signifies the maximum indentation depth at the end of the force-controlled holding phase. The resulting dimensionless C_IT_ value represents the percentage increase in indentation depth during the holding period. It provides insight into the material’s time-dependent mechanical properties and, for biological tissues like the cornea, can indicate fluid shifts within the tissue matrix.

### Corneal biomechanics under physiological conditions

To establish appropriate measurement condition for subsequent CXL investigation the biomechanical properties of five ex vivo corneal samples were evaluated under varying retrocorneal pressure conditions. The five corneoscleral discs were mounted in an artificial anterior chamber and pre-tensioned by adjustable retro-corneal fluid pressure (RCP). All measurements were performed in immersion with dextran-supplemented cell culture medium to ensure a comparable corneal thickness. NI was performed at 10 positions within the central 1 mm^2^ area of the cornea at five different RCP levels: 10, 15, 20, 30, and 40 mmHg. These pressure levels represent the physiological intraocular pressure range^24^ and extend to levels above that range. Following these measurements, the central 8 mm of each cornea was carefully excised (trephined), flatmounted to the base of a Petri dish using UHU SUPER GLUE (62687, UHU GmbH & Co. KG, Bühl, Germany) and covered with dextran-supplemented medium. These flatmounted samples were then subjected to the same measurement protocol, using 10 spatially resolved NIs. This flatmount method is comparable to previous NI studies^15–17,19^ allowing for direct comparison with existing literature. The consistent use of immersion conditions across all measurements ensures physiological hydration and comparability between the pre-tensioned and flatmounted sample fixation method.

### Riboflavin mediated corneal UVA-crosslinking

To quantify the effect of different riboflavin-mediated corneal collagen crosslinking protocols, human corneas were biomechanically characterized ex vivo in a spatially resolved manner by NI before (pre) and after (post) crosslinking. The indentation matrix used, which contained 28 indentations within the central 2 mm^2^ of the cornea, is shown in Fig. 1B. All tissue samples were saturated with a riboflavin solution by means of drops applied every 2 min for 30 min. To maintain corneal hydration and ensure sufficient riboflavin concentration during the entire procedure, riboflavin drops were continued every 2 min throughout the subsequent UVA irradiation. UVA exposure (370 nm wavelength, dose 5.4 J/cm^2^) was performed using a PLX 330 Platinum (Peschke Trade GmbH, Huenenberg, Switzerland) irradiation system. Throughout the crosslinking procedure and during NI measurements pre- and post-CXL, the RCP was maintained at 20 mmHg to ensure consistent testing conditions. Depending on the crosslinking protocol applied, one of three riboflavin solutions and one of five different UVA irradiance intensities were used. After crosslinking, the corneal hydration was equilibrated overnight in cell culture medium supplemented with 15% Dextran at a stable RCP of 20 mmHg to ensure comparable hydration for both NI pre- and post-CXL measurements. For each crosslinking protocol, 10 human corneal samples were used to ensure a robust dataset. This study encompassed two comparative analyses. The reference protocol (3 mW / cm^2^ for 30 min using 0.1% riboflavin + 1.1% Hydroxypropyl-methylcellulose (HPMC) (MedioCROSS® M)) served as the benchmark in both comparisons: first, when evaluating different riboflavin solutions, and second, when assessing various UV irradiation intensities. This approach ensured consistent comparisons across both experimental series.

To ensure spatial consistency between pre- and post-CXL measurements, all corneas remained mounted in the same artificial anterior chamber throughout the entire experiment. The chamber was fixed in a reproducible position relative to the nanoindenter using a custom sample mount. While exact repositioning cannot be guaranteed, we assume high consistency of central and peripheral measurement areas. Any minor deviations are considered negligible in relation to the treatment effects.

### Comparison of three different riboflavin solutions

For this comparison, CXL was performed using one of three commercially available riboflavin solutions with different osmolarities (Medio-Haus Medizinprodukte GmbH, Kiel, Germany): 0.1% riboflavin (MedioCROSS® H), 0.1% riboflavin + 1.1% HPMC (MedioCROSS® M), and 0.1% riboflavin + 20% dextran 500 (MedioCROSS® D). All CXL procedures used UVA radiation at an intensity of 3 mW / cm^2^ for 30 min, delivering a total dose of 5.4 J/cm^2^. Each riboflavin solution was used to crosslink 10 donor corneas, resulting in a total of 30 treated corneas for this comparison. Corneal thickness was measured at three time points using ultrasound pachymetry (Pachmate 2 DGH 55B Pachymeter, DGH Technology Inc., Exton, PA, USA): before CXL NI (T1), after CXL treatment (T2), and before post-CXL NI (T3). These measurements were taken to monitor the hydration status of the samples for NI. For biomechanical comparisons, we used the nanoindentation data acquired after T1 (before CXL) and after T3 (post-CXL, following overnight hydration equilibration), as these time points represent physiologically comparable hydration conditions.

### Comparison of five different UV radiation intensities

To compare the treatment effects of five different CXL protocols, including the standard Dresden Protocol and four accelerated versions, different UV irradiation intensities were used. The protocols employed were: 3 mW / cm^2^ for 30 min (Dresden Protocol, also used in the riboflavin solution comparison), 6 mW / cm^2^ for 15 min, 9 mW / cm^2^ for 10 min, 18 mW / cm^2^ for 6 min, and 30 mW / cm^2^ for 3 min. All CXL procedures in this comparison were performed using a 0.1% riboflavin + 1.1% HPMC solution (MedioCROSS® M). The Dresden Protocol served as the gold standard, while the other four protocols aimed to accelerate the treatment duration. Each of the five CXL protocols was applied to 10 corneas.

### Statistical analysis

To quantify the effects of different CXL protocols, we employed linear mixed effects models (LMEM) to account for the hierarchical structure of our data, where each cornea was characterized by multiple measurements both before (pre) and after (post) CXL treatment. Basing our statistical analysis on the method proposed by Herber and colleagues^25^, the analysis was structured around two key aspects of CXL treatment, each addressed by a separate statistical model:

- The first model compared the impact of riboflavin solutions of different osmolarity. It incorporated the specific riboflavin formulations as fixed effects and individual corneas as random variable.
- The second model examined the influence of different UV irradiation intensities, corresponding to various exposure times. This model used the different UV intensities as fixed effects, again with individual corneas as random variable.

In both LMEMs, pre-CXL (control) and post-CXL measurements were included for each protocol. To account for potential donor tissue variability, we included donor age, gender, and preservation time in tissue culture as additional fixed effects in our models. This approach allowed each model to estimate both the untreated biomechanical properties and the respective treatment effects for its specific focus (riboflavin solution or UV intensity), while controlling for donor-specific factors. Residual diagnostics, including Q-Q plots, were performed to assess normality of residuals and confirm that model assumptions were met. To assess measurement reliability, intraclass correlation coefficients (ICC) were derived from random-intercept models (response ~ 1 + (1|Cornea)). ICCs were first calculated for repeated NI measurements using two mounting approaches (flatmount vs. artificial anterior chamber) to evaluate the effect of tissue pretension on E_HZ_ reliability, based on 92 NI measurements from five samples. In a second analysis, baseline reliability was determined for E_HZ_ and C_IT_ from pre-CXL measurements under 20 mmHg pretension, including 1932 NI measurements from 70 samples. ICCs, defined as the proportion of between-cornea variance relative to total variance, quantify the consistency of repeated measurements. All statistical and graphical analyses were conducted using R statistical software^26^, with R Studio^27^ as the integrated development environment.

## Results

### Biomechanical properties of pre-tensioned corneal tissue

**Fig. 3:**
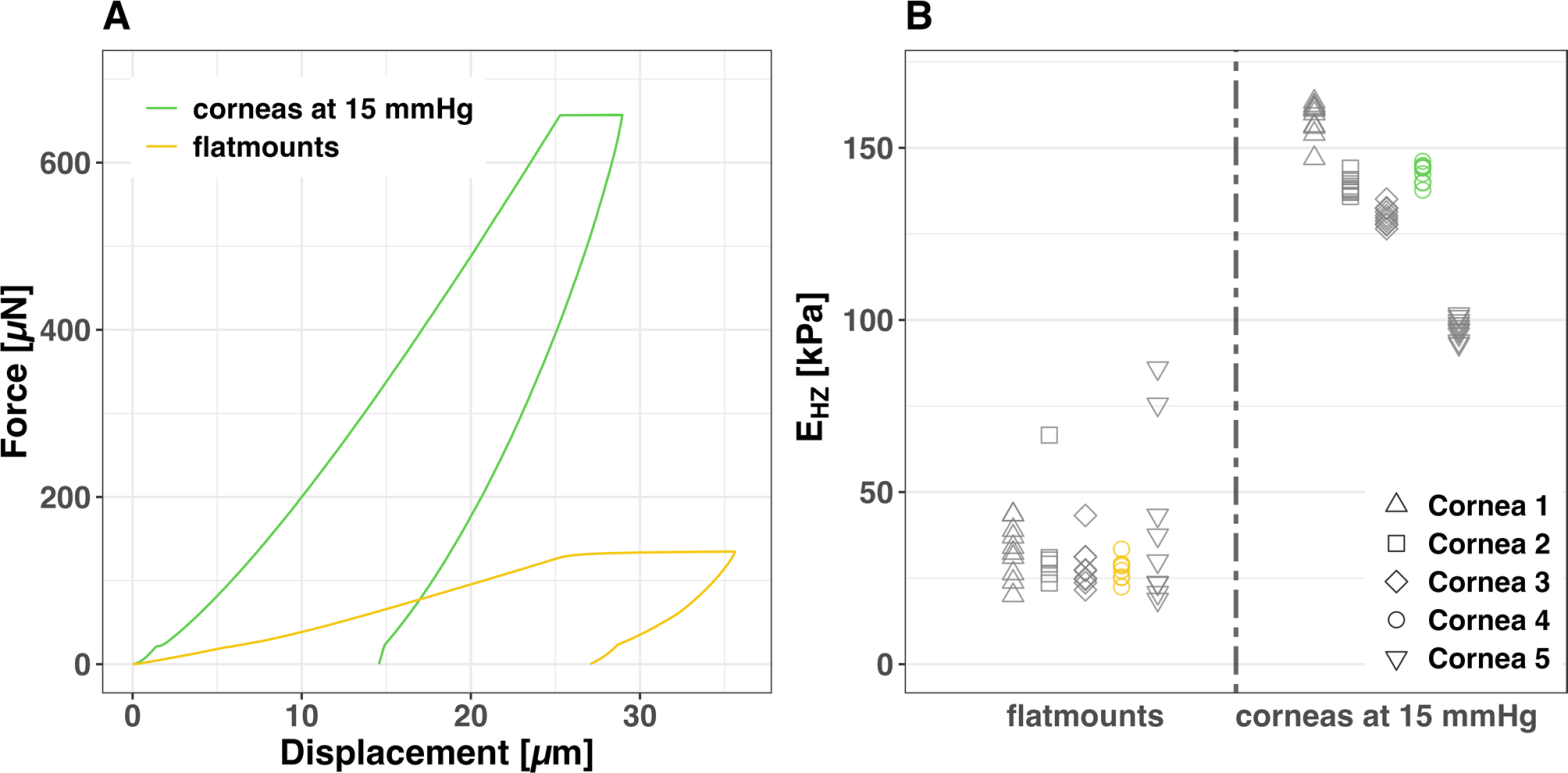
Force–Displacement-Diagram showing the mean progression curves of Nanoindentations of Cornea 4 at 15 mmHg retrocorneal fluid pressure (RCP) and tissue flatmounts (**A**). Scatterplot with illustration of the absolute E_HZ_ values of the measurements at 15 mmHg RCP and tissue flatmounts (**B**). The measured values of the different corneas are shape coded. The values belonging to the Force–Displacement-Curves (**A**) are also color-coded.

This study investigated pre-tensioned human corneal tissue biomechanics using five male donor samples (mean age: 83.2 ± 8.73 years; mean preservation time in tissue culture: 49.2 ± 5.97 days). Human corneal tissue in its physiological shape pre-tensioned by an RCP exhibits higher E_HZ_ values and a reduced intraindividual standard deviation compared to tissue flatmounts. Biomechanical differences between individual corneas become clearly detectable in the presence of physiological RCP (Fig. 3; Table 1). Reliability analysis of repeated measurements confirmed these findings. Flatmounts showed very poor reliability (ICC = 0.02), whereas mounting in an artificial anterior chamber under 15 mmHg pretension achieved excellent reliability (ICC = 0.98).

**Table 1:** E_HZ_ Statistics for Individual Corneas: Pre-tensioned at 15 mmHg vs. flatmounted.

Sample	Method	Median	Mean	SD
Cornea 1	flatmount	33.15	33.05	7.970
	15 mmHg	160.48	158.33	5.037
Cornea 2	flatmount	29.68	34.45	15.963
	15 mmHg	138.03	138.80	2.383
Cornea 3	flatmount	27.20	28.34	6.442
	15 mmHg	129.93	130.21	2.654
Cornea 4	flatmount	27.88	27.53	3.321
	15 mmHg	144.15	142.81	2.735
Cornea 5	flatmount	29.91	39.86	24.620
	15 mmHg	97.55	97.58	2.841

**Fig. 4:**
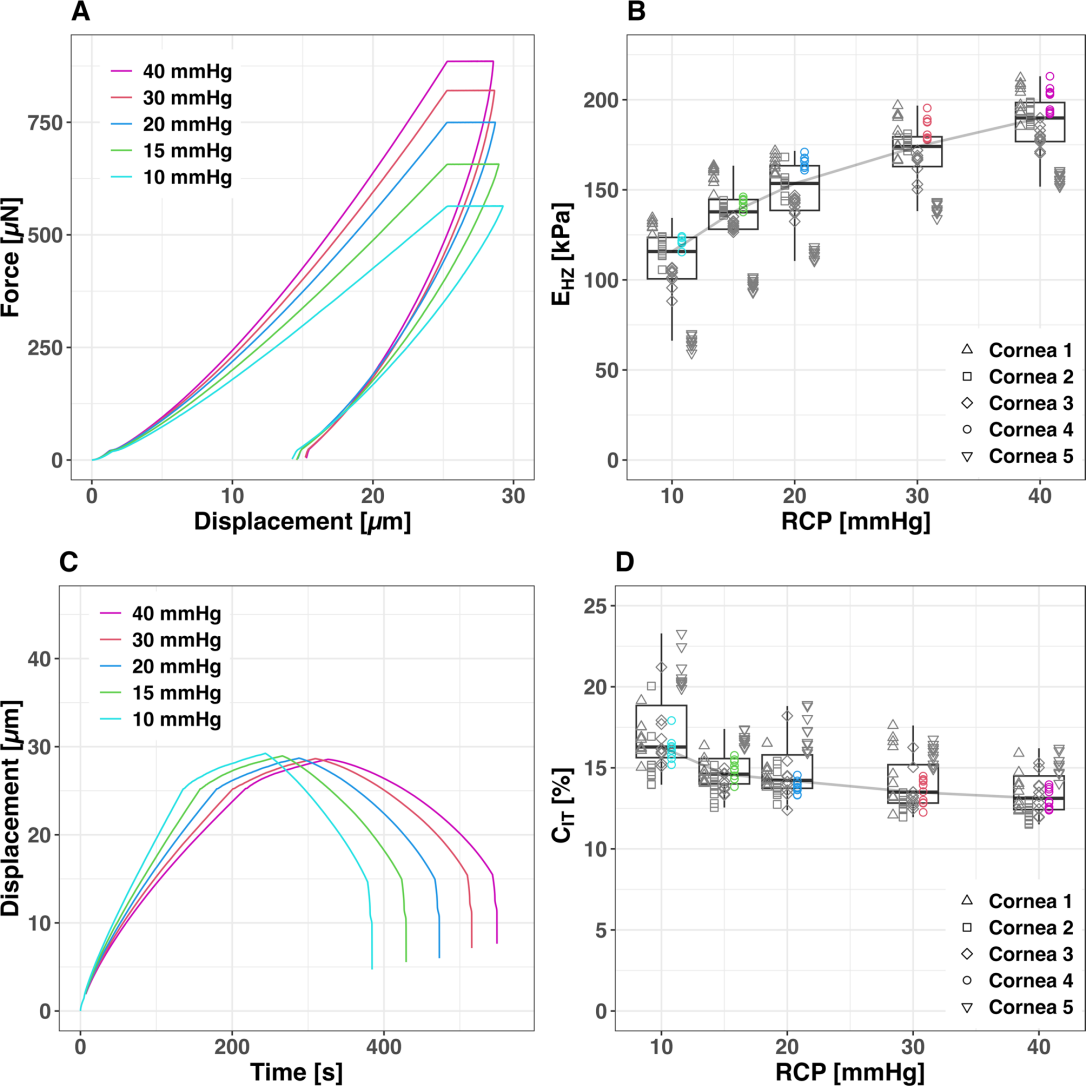
Force–Displacement-Diagram (**A**) and Displacement–Time-Diagram (**C**) showing the mean progression curves of Nanoindentations (Nis) of Cornea 4 at five different retrocorneal fluid pressure (RCP) levels. Boxplots with illustration of the absolute values and curve of the median E_HZ_ (**B**) and C_IT_ (**C**) over the five measured RCP levels. The boxplot displays median values (horizontal lines within boxes) and interquartile ranges (boxes). The measured values of the different corneas are shape coded. The values belonging to the Force–Displacement-Curves (**A**) and Displacement–Time-Diagram (**C**) and are also color-coded.

Increasing RCP levels generally lead to higher E_HZ_ and diminished C_IT_ values. However, these changes are not linear, as shown in (Fig. 4). For instance, in the 10–15 mmHg RCP range, E_HZ_ increases by 4.4 kPa / mmHg. In contrast, at higher pressures between 30 and 40 mmHg RCP, the E_HZ_ increase rate declines to 1.6 kPa / mmHg, illustrating the non-linear nature of corneal response to pressure changes (Table 2).

**Table 2:** Aggregated E_HZ_ and C_IT_ Statistics: Multiple RCP Levels vs. flatmounts.

	E_HZ_ [kPa]			C_IT_[%]		
Method	Median	Mean	SD	Median	Mean	SD
10 mmHg	115.75	107.17	23.36	16.28	17.22	2.35
15 mmHg	137.76	133.55	20.61	14.61	14.86	1.22
20 mmHg	153.54	147.89	19.32	14.23	14.84	1.58
30 mmHg	174.09	169.18	17.61	13.50	14.10	1.54
40 mmHg	189.88	185.88	17.74	13.12	13.47	1.30
flatmounts	29.06	32.65	13.94	38.39	38.16	9.37

### Quantification of the effect of different crosslinking protocols

This study compared seven different CXL protocols using 70 human donor corneas (mean donor age 74.8 ± 11.9 years, range 37 – 93 years; 16 females, 54 males), with a mean preservation time in tissue culture from tissue donation to the experiment of 50.29 ± 21.07 days. Baseline reliability of pre-CXL measurements under 20 mmHg pretension was excellent for E_HZ_ (ICC = 0.95) but only moderate for C_IT_ (ICC = 0.74).

### Effects of riboflavin osmolarity on corneal thickness and biomechanics

**Fig. 5:**
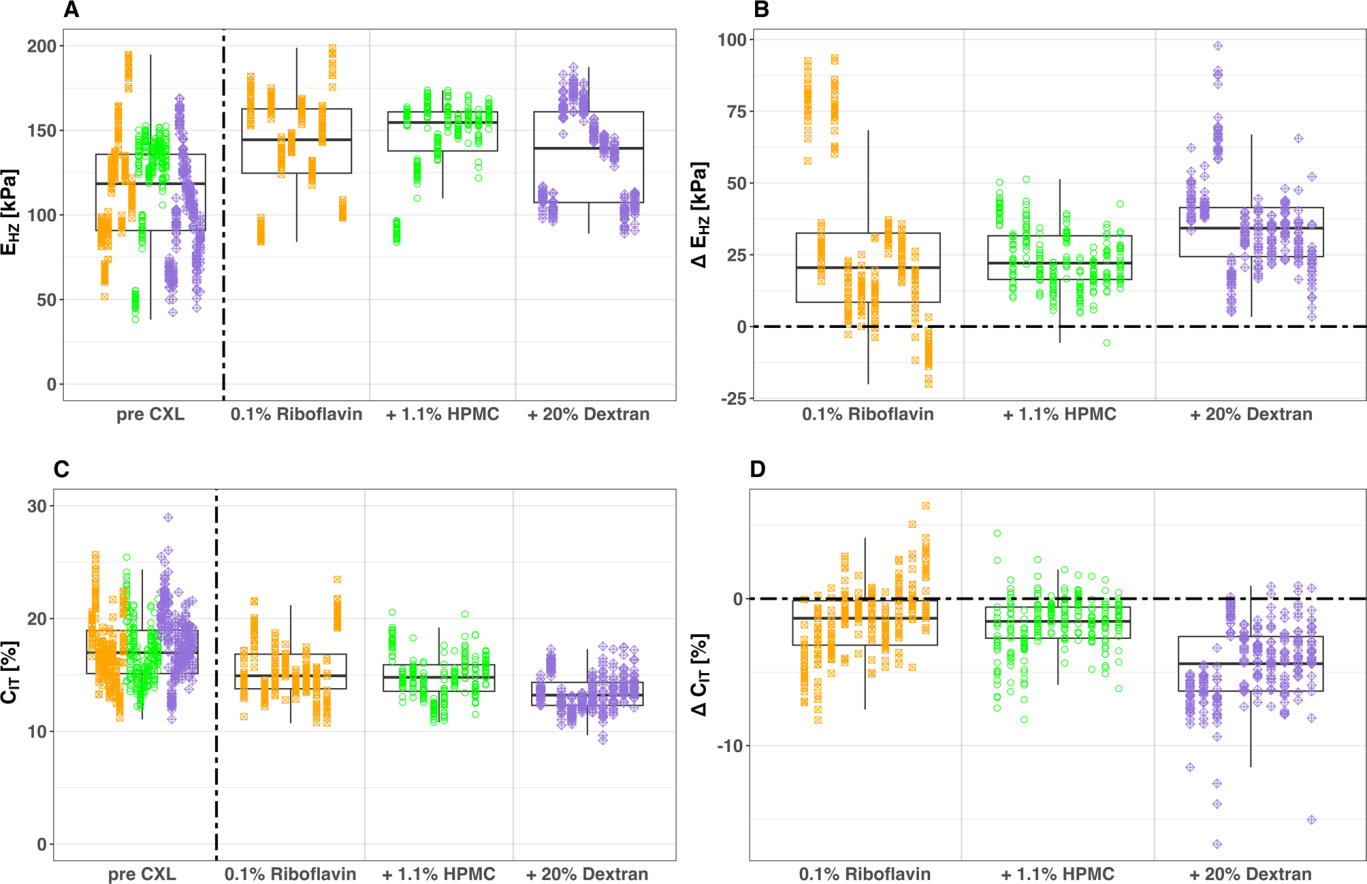
Boxplots of absolute values E_HZ_ (**A**) and C_IT_ (**C**) before and after corneal crosslinking (CXL) with one of three different Riboflavin solutions. The boxplot displays median values (horizontal lines within boxes) and interquartile ranges (boxes). The CXL effects are also visualized as changes Δ E_HZ_ (**B**), and Δ C_IT_ (**D**) using boxplots. CXL protocols shape and color-coded. All CXL treatments comparing riboflavin solutions were performed with a UV irradiation intensity of 3 mW / cm^2^ with an irradiation time of 30 min.

**Fig. 6:**
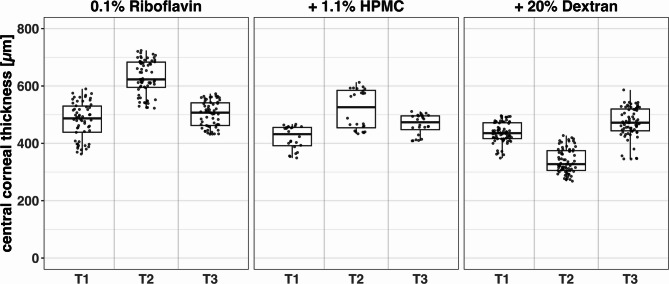
Boxplots of central corneal thickness measured by ultrasound pachymetry at three time points: before corneal crosslinking (CXL) (T1), immediately after CXL treatment (T2), and after overnight equilibration of tissue hydration state, before post-CXL Nanoindentations (T3). The plot compares results from CXL treatments using three riboflavin solutions of different osmolarity: 0.1% Riboflavin (9 corneas), 0.1% Riboflavin + 1.1% HPMC (4 corneas), and 0.1% Riboflavin + 20% Dextran (10 corneas). Boxplots represent the interquartile range (IQR) with a horizontal line indicating the median, while individual data points appear as dots.

The descriptive visualization in Fig. 5 illustrates measured values as absolute E_HZ_ and C_IT_ values as well as changes induced by CXL treatment (Δ E_HZ_ and Δ C_IT_) which are calculated by subtracting pre CXL from post CXL measurements. The absolute pre- and post-CXL values were used to construct the linear mixed-effects models (LMEMs). When including preservation time, donor age, and gender as covariates, only preservation time showed a statistically significant effect (*p* = 0.02). For the final analysis, we consequently reduced the model to include only preservation time, which remained significant (*p* = 0.018), showing a positive correlation with E_HZ_ (increasing by 0.55 kPa per day) and a negative correlation with C_IT_ (decreasing by 0.037% per day). The analysis calculated treatment effects for three riboflavin solutions: 0.1% riboflavin, 0.1% riboflavin + 1.1% HPMC, and 0.1% riboflavin + 20% dextran 500. Detailed results are presented in Table 3, providing a nuanced analysis accounting for preservation time in tissue culture variability. Baseline values were estimated for zero preservation days, enabling a precise estimation of treatment effects while accounting for variability due to preservation time of the corneal tissue in cell culture. Ultrasound pachymetry measurements (n = 23) revealed different effects on corneal thickness depending on which Riboflavin solution was used for the treatment. Corneas treated with pure 0.1% riboflavin (n = 9) increased in thickness by an average of 149.75 µm. Those treated with 0.1% riboflavin + 1.1% HPMC (n = 4) showed less swelling, increasing by 98.88 µm. In contrast, corneas treated with 0.1% riboflavin + 20% dextran (n = 10) decreased in thickness by about 98.77 µm. These measurements are represented in Fig. 6 and summarized in Table 4. From the initial 10 samples per group, different numbers had to be excluded from analysis due to technical limitations during pachymetry measurements.

**Table 3:** LMEM Results: Estimated Baseline E_HZ_ and C_IT_ Values pre CXL and Estimated Treatment Effects for Different Riboflavin Solutions post CXL.

Condition	E_HZ_ [kPa]			C_IT_ [%]			
	Estimate	Std.error	*p* value	Estimate	Std.error	*p* value	
Pre CXL	88.37	10.97	< 0.01	18.83	0.79	< 0.01	
0.1% riboflavin	27.64	0.93	< 0.01	− 1.52	0.13	< 0.01	**Δ CXL**
0.1% riboflavin + 1.1% HPMC	23.82	0.92	< 0.01	− 1.77	0.13	< 0.01	
0.1% riboflavin + 20% dextran	35.07	0.92	< 0.01	− 4.49	0.13	< 0.01	
Presevation time	0.55	0.22	0.02	− 0.037	0.16	0.03

**Table 4:** Central Corneal Thickness: Comparative Analysis of Three Riboflavin Solutions with Varying Osmolarity at pre-CXL (T1), Immediate post-CXL (T2), and post-CXL biomechanical characterization after thickness equilibration (T3) Timepoints.

Riboflavin solution	Timepoint	Number of successful measurements	Median	Mean	SD
0.1% riboflavin	T1	58	487.00	480.33	63.77
	T2	64	623.00	630.08	59.34
	T3	58	507.00	501.74	44.58
0.1% riboflavin + 1.1% HPMC	T1	24	432.00	420.25	40.81
	T2	24	526.00	519.13	68.88
	T3	24	473.50	466.04	35.39
0.1% riboflavin + 20% dextran	T1	70	435.50	437.36	35.47
	T2	70	327.50	338.59	43.12
	T3	70	472.00	473.04	53.19

### Effects of UV irradiation intensity on corneal biomechanics

**Fig. 7:**
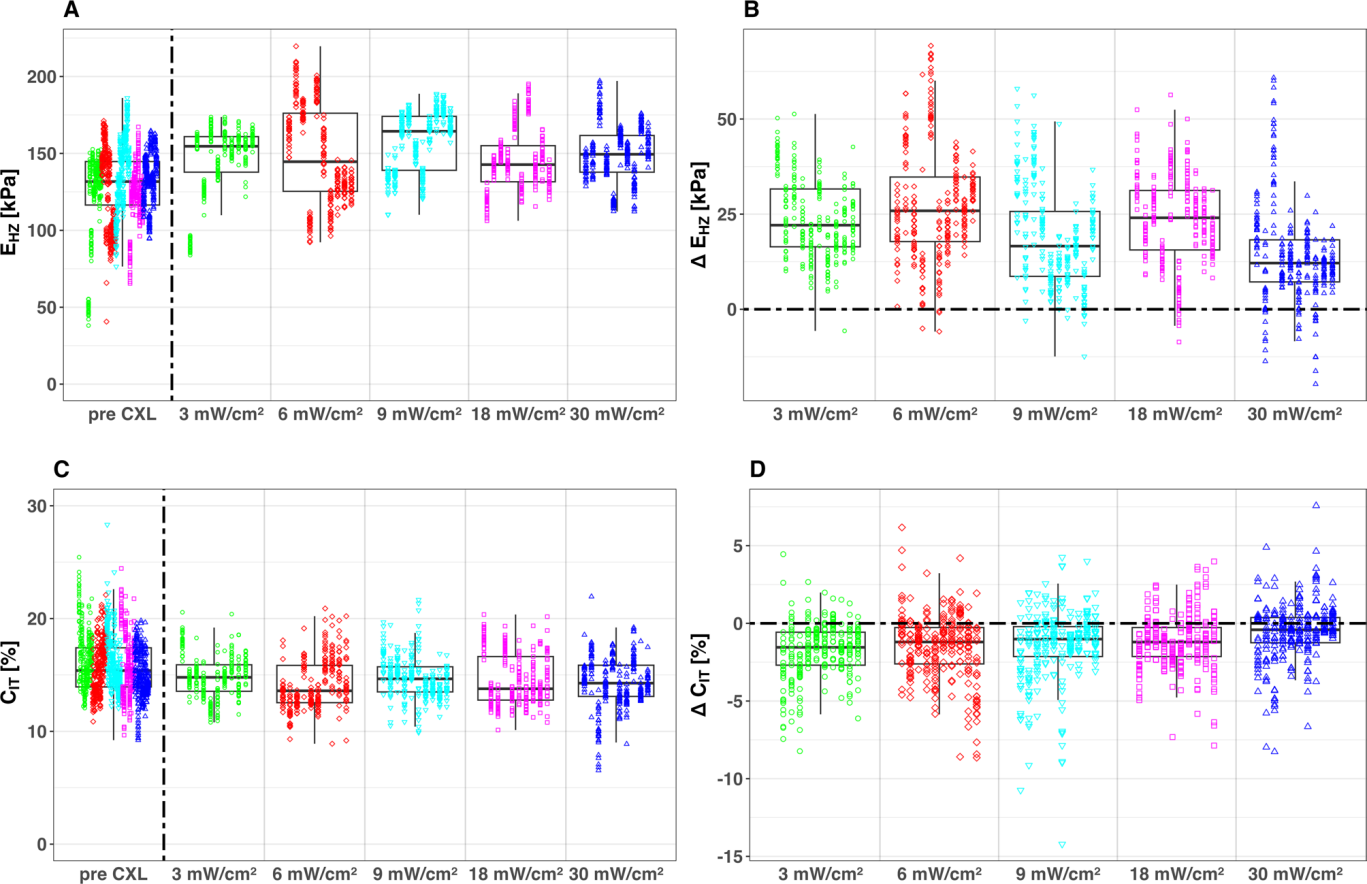
Boxplots of absolute values E_HZ_ (**A**) and C_IT_ (**C**) before and after corneal crosslinking (CXL) with one of five different UV irradiation intensities. The boxplot displays median values (horizontal lines within boxes) and interquartile ranges (boxes). The CXL effects are also visualized as changes induced by CXL Δ E_HZ_ (**B**), and Δ C_IT_ (**D**) using boxplots. CXL protocols shape and color-coded. For comparison of UV irradiation intensities, CXL was performed with an 0.1% Riboflavin + 1.1% HPMC solution (MedioCROSS M®).

The measured E_HZ_ and C_IT_ values, presented descriptively in Fig. 7 as absolute results and derived changes (Δ E_HZ_ and Δ C_IT_, calculated as post-CXL–pre-CXL), served as the basis for the LMEM analyses. These models evaluated the effects of different UV irradiation intensities (3, 6, 9, 18, and 30 mW / cm^2^) on corneal biomechanics, with UV irradiation intensity as a fixed effect and cornea as a random variable. Models that included preservation time, donor age, and gender as covariates showed no significant improvement in explaining the data compared to the simple model. Therefore, these factors were excluded from the final analysis. A detailed summary of the model estimates E_HZ_ and C_IT_ is provided in Table 5. The LMEM analysis showed statistically significant treatment effects (*p* < 0.001) for all CXL protocols in both comparisons.

**Table 5:** LMEM Results: Estimated Baseline E_HZ_ and C_IT_ Values pre CXL and Estimated Treatment Effects for Different UV Irradiation Intensities post CXL.

Condition	E_HZ_ [kPa]			C_IT_ [%]			
	Estimate	Std.error	*p* value	Estimate	Std.error	*p* value	
pre CXL	128.21	32.25	< 0.01	15.82	0.27	< 0.01	
3 mW/cm^2^	23.75	0.70	< 0.01	− 1.75	0.12	< 0.01	**Δ CXL**
6 mW/cm^2^	27.52	0.71	< 0.01	− 1.52	0.12	< 0.01	
9 mW/cm^2^	18.74	0.70	< 0.01	− 1.44	0.12	< 0.01	
18 mW/cm^2^	23.52	0.70	< 0.01	− 1.19	0.12	< 0.01	
30 mW/cm^2^	13.77	0.70	< 0.01	− 0.58	0.12	< 0.01	

## Discussion

This study provides a comprehensive assessment of corneal biomechanical properties under various conditions ex vivo, comparing human corneal tissue measured in a flatmounted configuration as previously published and under pre-tension. The latter novel approach allows for quantitative investigation of corneal biomechanics under near-physiological conditions, offering new insights into the effects of tissue pre-tension and various crosslinking (CXL) protocols.

The results of this study demonstrate that RCP-induced pre-tension has a major, non-linear impact on corneal E_HZ_ and C_IT_, with measurements at 15 and 20 mmHg pretension showing excellent reliability (ICC = 0.95 and 0.98) compared to poor reliability in flatmounted tissue (ICC = 0.02). Flatmounted tissue lacking pre-tension showed substantially lower E_HZ_ compared to measurements at 15 mmHg RCP, with readings differing significantly in both values and variation. The application of RCP revealed distinct E_HZ_ value clusters for individual corneas, highlighting interindividual differences. The mean modulus measured in native, flatmounted human corneal tissue (32.65 ± 13.94 kPa) is consistent with previously reported values from nanoindentation studies, including ~ 43 kPa by Nohava et al.^17^ and ~ 49 kPa by Ross et al.^19^. Despite methodological differences such as loading mode and rate, this agreement supports the reliability of our measurements and validates the baseline stiffness range observed in our setup.

The observed non-linear response to RCP has significant implications for measuring intraocular pressure and assessing corneal biomechanics clinically. This finding suggests a need to reassess current methodologies, as it may impact the precision of tonometry and the analysis of corneal topography, particularly in conditions such as keratoconus. Previous research has shown that corneal biomechanical properties, can significantly affect the accuracy of different tonometry devices^28^. Munkwitz and colleagues (2008)^29^ observed that while IOP measurement variations remained stable within the 7–22 mmHg range, they nearly doubled in magnitude for pressures between 23 and 60 mmHg. While measurement precision at pressures beyond the physiological range (e.g., above 30 mmHg) may be of limited clinical relevance, understanding the complex material properties of the cornea under various pressure conditions remains important. This insight highlights the significance of considering the cornea’s natural pre-tension in biomechanical characterization. Consequently, assessment methods that disregard the eye’s physiological tension may provide only limited clinically valuable information. Recent advances in corneal biomechanics modeling offer promising avenues for clinical application of our findings. Nambiar et al.^11^ developed accurate numerical models of porcine corneas based on ex vivo measurements, while Issarti et al. highlighted the importance of internal ocular structures in human corneal deformation through finite element analysis. These studies demonstrate that finite element modeling can bridge the gap between ex vivo absolute force measurements and in vivo biomechanical assessment techniques. Our experimental model, which measures corneal biomechanical properties under physiological conditions, provides essential data to calibrate and validate these finite element models. This integration of experimental and computational approaches could enable the development of more accurate patient-specific corneal models, potentially facilitating personalized treatment strategies and improving outcome predictions for ophthalmic interventions.

Our study further demonstrates that all CXL methods significantly increase E_HZ_ and reduce C_IT_, with different magnitudes depending on the specific protocol used. We observed enhanced effects with hyperosmolar riboflavin and diminished effects with high-intensity, short-duration UV radiation.

Regarding riboflavin osmolarity, we found that hyperosmolar solutions containing dextran led to corneal thinning, potentially enhancing cross-linking in thick corneas. Conversely, hypoosmolar solutions cause corneal swelling, theoretically allowing treatment of thin corneas. However, our results, supported by a case report from Hafezi^30^, raise concerns about CXL efficacy in swollen thin corneas. The case report decribes CXL failure in an extremely thin cornea (268 μm) despite successful swelling to over 400 μm, with subsequent keratoconus progression of 2.3 diopters at 6 months post-CXL. The use of hypo-osmolar solutions enables the treatment of thinner corneas. However, it appears that the efficiency of the treatment and the stiffening effect on the tissue may decrease when corneas are significantly swollen before the procedure. This suggests that a minimal preoperative stromal thickness (around 330 μm) may be crucial for successful CXL, and excessive corneal swelling should be approached with caution. These treatment effects were confirmed both by visual analysis of absolute changes (Δ E_HZ_ and ΔC_IT_) in Fig. 5 and by the linear mixed-effects model (Table 3), which accounts for inter-individual variability and baseline stiffness. The model results support the observed trends, including enhanced stiffening with hyperosmolar riboflavin and reduced effects with high-intensity UV protocols.

While cross-linking was measurable across all UV intensities tested, efficacy varied notably. High-intensity, short-duration protocols (particularly 30 mW / cm^2^ for 3 min) showed reduced effects compared to lower-intensity, longer-duration treatments. This aligns with previous findings of decreased corneal stiffening at higher UV intensities^31,32^. Our results suggest that very short irradiation times (under 5 min) may compromise treatment efficiency, indicating that the relationship between UV intensity and efficacy is more complex than the Bunsen-Roscoe law implies, likely influenced by factors such as oxygen availability and diffusion.

These findings have important clinical implications. Hyperosmolar riboflavin might benefit patients requiring maximal corneal stiffening, while accelerated, high-intensity protocols may not provide the same degree of biomechanical strengthening as standard protocols. The mechanisms underlying these differences, such as improved photosensitizer penetration with hyperosmolar riboflavin and oxygen availability during high-intensity protocols, warrant further investigation.

Several limitations should be acknowledged. The use of healthy donor corneas may not fully reflect the biomechanical properties of ectatic corneas typically treated with CXL. The potential influence of dextran in our experimental setup, while necessary for maintaining physiological corneal hydration, may affect the absolute values of our measurements. Tissue bending during NI may also introduce some variability. Finally, the use of the Hertz model, which assumes a flat, non-adhesive surface, represents a methodological limitation. We minimized curvature effects by indenting only to shallow depths (25 µm) at the relatively flat central corneal apex and performing measurements in a fluid medium which reduced adhesion force; however, minor influences from curvature and adhesion cannot be entirely excluded. Future studies could apply these methods to keratoconic corneas, explore alternative methods for maintaining physiological corneal hydration during testing, and investigate measurement techniques that further reduce potential sources of error.

This study provides valuable insights into corneal biomechanics under near-physiological conditions and the effects of various CXL protocols. Our findings illustrate the importance of considering tissue pre-tension in biomechanical assessments and offer quantitative data to guide the optimization of CXL treatments. Future research should focus on translating these ex vivo findings to in vivo applications and exploring their implications for clinical practice in corneal ectatic diseases.

## Data Availability

The datasets supporting this study can be obtained from the corresponding author upon a reasonable request.
